# Arsenic in the Treatment of Uterine Affections

**Published:** 1868-11

**Authors:** 


### ARSENIC IN THE TREATMENT OF UTERINE AFFECTIONS.

Arsenic, according to Waring and Stillé, has been used with decided benefit in carcinoma of the uterus, in irritable uterus, and in menorrhagia, by Dr. Hunt, of Dartmouth, England. Its value in atonic menorrhagia has been confirmed by the celebrated Dr. Locock, who states that he has employed it with great success in this and many other uterine affections. He thinks it acts specifically. In one case of cancer of the uterus, in which no relief was obtained from twenty-four grains of morphia, great ease and benefit accrued from small doses of Fowler's

Solution. Dr. Locock thinks highly of arsenic in this class of diseases. In menorrhagia, leucorrhœa, and uterine hemorrhage, in threatened abortion, and after delivery, Dr. A. Burns speaks of arsenic as a most reliable remedy. In leucorrhœa he gives five drops of Fowler's Solution three times a day, till a cure is effected. Dr. Simpson has used it successfully in amenorrhœa and dysmenorrhœa, as well as in that peculiar affection of the bowels characterized by copious discharges of membranous shreds, and accompanied by great emaciation and a long train of neuralgic and other nervous symptoms.

M. Imbert Gourbeyre has drawn attention to the great utility of Fowler's Solution in the treatment of vulvar pruritus. Other physicians have found it more useful in ulcerations of the os uteri than the application of nitrate of silver and other caustics. In fact, it is as beneficial in various ulcerations of the mucous membranes as it is well known to be in many ulcerations and eruptions upon the skin.

As regards the *modus operandi* of arsenic, it may be put down as a tonic when given in small or medicinal doses. It then excites a sense of warmth in the stomach and bowels, increases the appetite, and in some degree also the fæcal and urinary discharges. The skin becomes warmer, the pulse fuller and more frequent, the muscular system more active, and the whole organism invigorated, freer and lighter in its movements, and even the mind improves in activity and power. Such a remedy is well calculated to remove chronic congestions, and even chronic inflammations, such as depend upon a feeble rather than a sthenic condition of the vascular and uterine system.

In larger doses it acts as an irritant, like pepper, mustard, ginger, and may unquestionably stimulate the stomach and promote the action of the bile, pancreas, and mucous glands, and thus favor nutrition.

The somewhat fanciful Germans suggest that it may act not only as a tonic, or rather stimulant, but as a delicate caustic to new formations and those with fragile attachments. Others say that the long-continued use of arsenic causes chronic anæmia, coupled with disintegration of the blood corpuscles, diminution of the proportion of fibrin, and general impairment of the vital principle upon which the normal qualities of the blood depend. Hence they infer that it acts as an antagonistic agent against hyperæmia, congestion, chronic or subacute inflammation, etc. In fact, they declare that it is the most certain and powerful emmenagogue of the whole Materia Medica. The typical cases of amenorrhœa in which its use is most appropriate, are thus described by Vogt:—They occur in persons of a torpid and relaxed constitution, and disposed to mucous accumulations and menorrhœal discharges; in other words, there is a general atony of the system in which the uterus participates, and of which the capital sign is leucorrhœa occurring exclusively, or in an aggravated degree about the catamenial period. When there is a great want of tone in the system it may have to be aided with bark, iron, and other tonic medicines. It is thought to be almost as specific against uterine leucorrhœa as copaiba is against gonorrhœa, and to act in a somewhat similar manner. Kopp has recommended it in *sterility* arising from uterine leucorrhœa, or depending upon a torpid state of the sexual organs. He also states that it acts as an excitant of the venereal propensity.

It is very easy to understand the action of sabina in these disorders, and to expect and anticipate the benefit which will almost surely follow its use. But Kopp has recommended it in more complicated states, such as *dysmenorrhœa*, particularly in unmarried females, and when it is attended with expulsive pains and discharges of scanty, dark and clotted blood, or when, as in other cases, there is an augmented flow taking place irregularly, ceasing, then reäppearing, etc. He usually prescribed it in conjunction with borax, and states that it relieves *menorrhagia* when this acts in congestive uterine affections in the same advantageous way that it does in plethora, in the determination of blood to the head, and in apoplectic congestions, against which Dr. L. Piquot considers that it operates by reducing in a remarkable manner the excess of the red globules of the blood, which, in these cases, he supposes to exist in a morbid and dangerous degree, having witnessed more relief from the use of liquor arsenicalis in combination with liquor potassæ, than from the local abstraction of blood, blisters, setons, etc.—*Medical Gazette.*

---

Inhalation of Oxygen after Tracheotomy.—Dr. Jacobi, of New York (*Medical Record*), after opening the trachea in a case of croup, finding the patient to suffer much from difficult respiration, resorted to the inhalation of oxygen gas mingled with common air, and introduced through the canula by an elastic tube. The result was very satisfactory. The child became quiet, and was finally so well satisfied with it that he would not allow the tube to be withdrawn. The relief, however, was transient. Next morning convulsions came on, but as soon as the oxygen was inhaled they ceased. The child finally died.

